# Improving asthma case detection among children and adolescents through clinic-based screening in primary care health facilities in Uganda: a cluster randomised trial

**DOI:** 10.1136/bmjresp-2025-003368

**Published:** 2026-04-17

**Authors:** Rebecca Nantanda, Andrew Ssemata Sentoogo, Sarah Namusoko, Phiona Ekyaruhanga, Mary Goretty Kuteesa, Fred Matovu, Levicatus Mugenyi, Jonathan Grigg

**Affiliations:** 1 Makerere University Lung Institute, College of Health Sciences, Makerere University, Kampala, Uganda; 2 Department of Psychiatry, College of Health Sciences, Makerere University, Kampala, Uganda; 3 MRC/ UVRI & LSHTM Uganda Research Unit, Entebbe, Uganda; 4 School of Economics, College of Business and Management Sciences, Makerere University Kampala, Kampala, Uganda; 5 Centre for Genomics and Child Health, Blizard Institute, Barts and The London School of Medicine and Dentistry, Queen Mary University of London, London, UK

**Keywords:** Asthma in primary care, Paediatric asthma

## Abstract

**Introduction:**

Underdiagnosis of asthma in children and adolescents is a major challenge, particularly in low-resource settings. We aim to assess the clinical and cost-effectiveness of screening for asthma symptoms among children and adolescents with respiratory symptoms presenting at primary care health facilities in Uganda. The feasibility and acceptability of routine screening will also be explored.

**Methods and analysis:**

A cluster-randomised trial, using screening for asthma symptoms as the intervention, will be conducted in health centres in Jinja region, Eastern Uganda. We hypothesise that screening for asthma symptoms at clinical care points will lead to an increase in the proportion of children diagnosed with asthma. The health centres will be randomised into intervention and control arms. In the intervention sites, 1050 children aged 2 months to 17 years with respiratory symptoms will be screened for asthma symptoms using the International Study on Asthma and Allergies in Children verbal questionnaire. History and physical examination will be conducted among the screen positives to identify those with asthma. Data on asthma diagnoses 12 months before the intervention and during the intervention will be collected from both the control and intervention health centres. Data on direct and indirect costs of screening will be collected prospectively. Focus group discussions (FGDs) will be conducted among health workers and key informant interviews (KII) among facility in-charges at the intervention sites to assess the feasibility and acceptability of the intervention. The primary outcome will be the proportion of asthma diagnoses. A random-effects logistic regression model, adjusting for baseline data while accounting for facility level clustering, will be used to assess the effectiveness of screening. Cost-effectiveness will be assessed by computing the incremental cost-effectiveness ratio. Framework analysis will be used to analyse data from the FGDs and KIIs.

**Ethics and dissemination:**

Ethics approval was obtained from Makerere University School of Public Health Research and Ethics Committee (SPH-2024-552), Uganda National Council for Science and Technology (HS4136ES) and the Queen Mary University of London Research Ethics Committee in the UK (QME24.0514). All participants will provide written informed consent. Children aged 8 years and above will provide assent, in addition to consent by their parents/caregivers. The study results will be disseminated through publications in peer reviewed journals, conferences, participant feedback meetings and Makerere University Lung Institute website. Policy briefs will be shared with key stakeholders including the Ministry of Health and Jinja District/City administrators.

**Trial registration number:**

ISRCTN16018011.

**WHAT IS ALREADY KNOWN ON THIS TOPIC**

**WHAT THIS STUDY ADDS**

- This study will provide information on the feasibility, acceptability and clinical and cost-effectiveness of symptom-based screening for asthma in clinical care settings, in improving diagnosis of asthma in children and adolescents in Uganda and similar settings globally.

**HOW THIS STUDY MIGHT AFFECT RESEARCH, PRACTICE OR POLICY**

- This study will provide new and valuable empirical quantitative and qualitative evidence on the importance of screening for asthma in every child and adolescent who presents with respiratory symptoms in health centres in Uganda. This evidence will be critical in informing clinicians, healthcare planners and policymakers in implementing and scaling up clinic-based screening for asthma symptoms to address the high prevalence of undiagnosed asthma in children and adolescents in low-income settings.

## Introduction

 Asthma is the most common chronic disease in children and adolescents but is underdiagnosed, especially in low and middle-income countries.^1^,^3^ Studies show that more than half of children with asthma symptoms are not diagnosed or are misdiagnosed as pneumonia and pulmonary tuberculosis.^1 4^ A recent study among children aged 12–14 years in six African countries showed that more than two-thirds of the children with asthma had severe symptoms and the majority (77.9%) had never been diagnosed.^5^ A study in Nicaragua among 199 children aged 2–17 years showed that 65 (32.7%) of them had asthma symptoms; however, 51 of them had not been previously diagnosed, yielding an underdiagnosis rate of 78.5%.^6^ In Nigeria, more than half of rural school children with asthma symptoms had never been diagnosed and therefore were not receiving any asthma treatment.^7^ Undiagnosed and therefore untreated asthma increases the risk of chronic obstructive pulmonary disease, is associated with high healthcare costs, missed workdays for caregivers, psychosocial stress and poor quality of life for the affected children and their caregivers.^8^,^12^

The reasons for underdiagnosis and/or misdiagnosis of asthma in children are diverse and include under-reporting of symptoms by patients and/or caregivers, poor access to care and poor diagnostic sensitivity of available tests like spirometry.^10 13^ Studies have also indicated that many healthcare providers do not routinely assess children with recurrent/chronic respiratory symptoms for asthma, partly due to high patient load and low knowledge about asthma, leading to missed opportunities for early diagnosis and treatment.^2 3^ Adherence to asthma guidelines also remains a major challenge, contributing to underdiagnosis and misdiagnosis of asthma in children.^14 15^

Health facility-based screening for asthma symptoms has the potential to improve early identification of children with asthma. The use of a standardised screening tool can help to identify those who require detailed assessment for asthma. However, the evidence on the effectiveness of such interventions is limited. A study in a primary care setting that employed screening for asthma symptoms in children indicated that this intervention identified 34.5% of children with undiagnosed asthma.^16^ In the USA, a hospital-based survey on undiagnosed and poorly controlled asthma in children ≥2 years presenting at the emergency unit found that 26% (282/1098) had probable asthma, with 23.7% (67/282) reporting no previous diagnosis of asthma.^17^ This protocol paper describes a study on assessment of the clinical and cost-effectiveness of screening for asthma symptoms among all children and adolescents with respiratory symptoms in primary healthcare facilities in Uganda. The feasibility and acceptability of routinely screening for asthma symptoms will also be explored.

### Study aims and objectives

The study aims to improve understanding of the potential clinical and economic benefits of screening for asthma symptoms for every child and adolescent with respiratory symptoms, towards early diagnosis of asthma in Uganda. The specific objectives of this cluster-randomised trial (CRT) are: (1) to evaluate the effectiveness of clinic-based screening for asthma symptoms among children and adolescents with respiratory symptoms in identification of patients with undiagnosed asthma, (2) to assess the cost-effectiveness of screening for asthma symptoms and (3) to assess the feasibility and acceptability of clinic-based screening for asthma symptoms among children and adolescents.

## Methods and analysis

### Study design and setting

The study will have two designs: CRT and cross-sectional designs. The CRT design will be used to assess the clinical and cost-effectiveness of clinic-based screening for asthma symptoms. The intervention will be screening for asthma symptoms in every child aged 2 months up to 17 years who presents with respiratory symptoms at the study sites, using the International Study on Asthma and Allergies in Children (ISAAC) verbal questionnaire. The cross-sectional design will be used to assess the feasibility and acceptability of clinic-based screening for asthma symptoms, employing qualitative data collection methods. The study will be conducted in eight primary care health facilities in Jinja city and the district local government (DLG), in Eastern Uganda. Jinja city was carved out of Jinja district in 2022. The population of the city and DLG is 660 089 and an estimated 48.7% (283 305) are below 18 years of age. About 42.5% live in the city.^18^ The city and DLG have primary care settings of interest (health centre (HC) III, IV and a general hospital), and a regional referral hospital (secondary care) where patients that require specialist assessment are referred. HC IVs and HC IIIs offer general outpatient and inpatient care for medical conditions. HC IVs, in addition to medical care, offer emergency obstetric care. There are 12 government HC IIIs, 5 HC IVs, 1 general hospital and 1 regional referral hospital. This setting is ideal for understanding the cascade of asthma care from primary care settings to the regional referral hospital.

### Study population

The target population will be children aged 2 months to 17 years presenting to HC IVs and HC IIIs with respiratory symptoms and their caregivers, as well as healthcare workers at the intervention sites.

### Sampling strategy and sample size estimation

**Figure 1:**
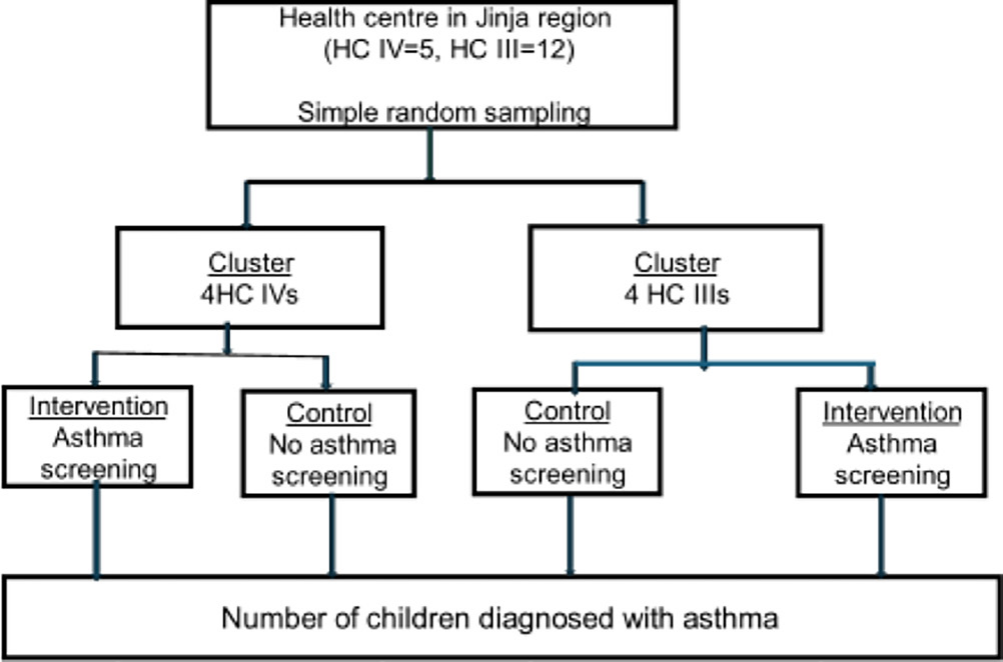
Summary of study procedures. HC, health centre.

Four HC IVs and four HC IIIs from the study area will be selected using simple random sampling. The selected health facilities will then be randomised to the intervention and control arms using simple randomisation in the ratio 1:1 leading to a total of four health facilities in the intervention (two HC IVs and two HC IIIs) and an equal number in the control arm (figure 1). The sample size determination is based on the study endpoint which is the proportion of patients diagnosed with asthma in each arm. A study in Brazil estimated 50% true positives from a screening tool.^19^ Based on this study, the estimated minimal sample size of the participants will be 2100 (ratio 1:1). With this sample, we anticipate diagnosing at least 15% more asthma cases in the intervention arm than in the control arm, assuming 80% power, 95% CI and a design effect of 1.5 to account for facility level clustering. The cost-effectiveness of screening for asthma symptoms in children and adolescents will be assessed using a randomly selected sample of 315 (30%) of the 1050 participants in the intervention arm. To assess the feasibility and acceptability of screening for asthma symptoms and perspectives on the implementation, focus group discussions (FGDs) comprising 8–12 health workers from the intervention sites will be conducted as well as the key informant interviews (KIIs) with facility in-charges from the intervention facilities.

### Eligibility criteria

Children: Children aged 2 months to 17 years presenting to the study sites for a medical visit and their parents/guardians will be eligible to participate in the study. We shall exclude those attending for other services, including disease prevention and health promotion services like immunisation, growth monitoring and reproductive health, and the unaccompanied. Children presenting after 17:00 and overnight will be excluded due to the challenges of having the research team on-site during these hours.

Healthcare workers: All health workers who are assigned to work in the study health facilities and are involved in the care of children, whether paid or volunteers, will be eligible. We shall exclude support staff like secretaries, security guards and drivers.

### Data collection and analysis

#### Data collection

Prior to data collection, all health workers in the intervention and control sites will be trained on diagnosis and management of asthma in children and adolescents. We shall use Global Initiative for Asthma (GINA) guidelines to develop the training materials and tools to highlight key features in diagnosis, management and care. All study sites will be provided with asthma medicines to support the diagnostic processes such as observing for response to short-acting beta agonists (SABA) for those that presented with exacerbations.

All children and adolescents attending the intervention study sites for clinical care will be screened for respiratory symptoms such as cough, difficulty in breathing and wheezing. Those found to be eligible (aged 2 months to 17 years, having at least one respiratory symptom), together with their parents/guardians, will be invited to participate in the study. The research assistant will provide information about the study to the potential participants, including study aims, study processes and ethical considerations, to the parents/guardians. Children aged 8 years and above will also be given the information as part of the process of obtaining assent. After obtaining written informed consent from the parents/guardians and assent (for children aged ≥8 years), the research assistant will administer the screening tool (ISAAC questionnaire). Children who will screen positive will undergo clinical assessment for asthma. Screen positives will be considered those children who will answer ‘Yes’ to the question ‘Has your child/have you had wheezing in the past 12 months?’ or/and ‘Has your child/have you ever had asthma?’. Clinical assessment for asthma will involve asking questions about chronic/recurrent cough, breathing difficulties, chest pain/tightness, shortness of breath, association of symptoms with triggers such as viral respiratory infections, smoke, dust, strong smells, physical exercise, extremes of weather, among others. A history of allergies in the participant such as allergic rhinitis, eczema and food allergies will be asked. In addition, participants/caregivers will be asked about family history of asthma. A physical examination will also be conducted. For children who will be found to have symptoms of an exacerbation such as wheezing, difficulty in breathing, shortness of breath, chest tightness, inhaled salbutamol, which is a SABA, will be administered using a spacer and in age-appropriate doses, and the response will be noted. A positive response will be considered if there is improvement in the symptoms such as disappearance of wheeze, reduction in respiratory rate and reduction or disappearance of chest in-drawing. Participants who will be found to have asthma following clinical assessment will be advised to continue to seek care for their asthma. The diagnosis of asthma will be made pragmatically (based on clinical assessment), because access to lung function testing (LFT) in the majority of HCs and hospitals in Uganda is still a big challenge^13 18^ and so, we shall mimic the real-life situation of diagnosing asthma based only on clinical assessment in the health facilities. We shall also assess asthma control using the ACT (Asthma Control Test—for children 12 years and above), childhood ACT for children aged 5–11 years and GINA guidelines for children 5 years and younger. Although there are tools that have been used for assessment of asthma control in children less than 5 years such as the Test for Respiratory and Asthma Control in Kids,^20^ this tool was developed in a high-income setting and has not been validated in low-income settings like Uganda. Instead, we have chosen to use the GINA asthma control assessment tool which is currently used in usual clinical care in Uganda. Participant enrolment will be done consecutively at all intervention study sites until the sample size of 1050 participants is realised. Data on costs of inputs for screening for asthma symptoms such as stationery and staff time will be collected prospectively throughout the data collection period. We shall collect data on the time taken to administer the screening tool on 315 (30% of 1050 participants in the intervention arm) randomly selected participants to assess the cost-effectiveness of the intervention.

After completion of enrolment, FGDs with the health workers will be conducted to explore their experiences and views on feasibility and acceptability of screening for asthma symptoms in children and adolescents presenting with respiratory symptoms at their health facilities. KIIs will also be conducted with the health facility in-charges. These interviews will provide insights into organisational and systemic factors that may influence adoption of the screening tool in routine clinical practice, complementing the health worker FGDs.

At the intervention and control sites, data on number of children and adolescents diagnosed with respiratory illness including asthma will be collected from the patient register once a week during the intervention period and entered in the database. We shall also extract the same type of data from patient registers in the 12 months prior to the start of the study in both intervention and control facilities.

### Data management and analysis

Descriptive statistics including percentages for categorical data and mean (SD) or median (IQR) for numerical data will be used to summarise participants’ baseline characteristics. The primary outcome will be the proportion of participants with asthma symptoms, calculated by dividing the number of screen positives by the total number enrolled. The proportion of asthma diagnoses will be presented as a percentage with 95% CI. Descriptive statistics will be used to analyse for the secondary outcomes of asthma symptom severity and control in the intervention arm. OR with 95% CI will be used to estimate the difference in asthma diagnoses attributable to screening for asthma symptoms between the intervention and control arms. We shall use a random-effects logistic regression model to estimate OR adjusted for baseline data while accounting for facility level clustering. Intention-to-treat analysis will be used. In addition, we shall analyse the performance of the screening tool among the intervention arm by estimating the positive predictive value (PPV). The PPV will estimate the percentage of persons that truly have asthma disease among those screened positive for asthma symptoms.

To assess the cost-effectiveness of clinic-based screening for asthma symptoms, we shall compute the incremental cost-effectiveness ratio from a health system perspective. Specifically, we shall compute the cost per additional diagnosis of asthma made and compare it with a predefined threshold like dollars per quality adjusted life years from published literature on similar interventions and settings, to judge if the additional cost of screening is a worthwhile investment for the extra health gain. The health system costs to consider will include staff training and staff time for administering the tool. The time horizon will be in line with the span of the data collection (12 months preintervention and time for the intervention). Sensitivity analysis will incorporate a longer time horizon and different age categories: 2 months−5 years, 6–12 years and 13–17 years.

Qualitative data from the FGDs and KIIs will be analysed using the framework analysis approach.^21^ Specifically, a coding framework will be developed to be used in coding data from the FGDs and KIIs. Each transcript will be coded by at least two researchers and assessed for consistency in coding. Any disagreements about the codes will be brought to the research team and discussed until agreement is reached. We shall use both manual and NVivo software for coding and analysis.

### Public and patient involvement

The public was not involved in the conception and design of the study. However, community leaders and stakeholders will be involved in the implementation of the study through the community advisory board, which will act as a link between the communities and participants, and the researchers. They will also actively participate in the dissemination of the study results.

### Data management

Quantitative data will be collected using hard copy case record forms (CRFs) and entered onto the REDCap platform (2022 Vanderbilt University). The data manager will run data quality checks periodically, including CRF completion. Data captured from the health facility patient registers from both the intervention and control sites will first be checked for quality by one of the research team members. Every month, a member of the research team will randomly select a proportion of the diagnoses captured, and using the source document (the patient register), physically verify if all the data was captured correctly. Further quality checks will also be performed by the data manager after entry onto the REDCap platform. Any irregularities in the data will be presented during the weekly research team meetings where corrective actions will be discussed and recommendations made.

Qualitative data will be collected using audio-recorders and field notes. No personal information will be entered onto REDCap or audio-recorders.

A dedicated data management team will continuously monitor the data as part of the data quality assurance process and address irregularities in a timely manner.

## Ethics and Dissemination

Ethics approval has been obtained from Makerere University School of Public Health Research and Ethics Committee (SPH-2024-552), Uganda National Council for Science and Technology (HS4136ES) and the Queen Mary University of London Ethics of Research Committee in the UK (QME24.0514). All participants will provide informed consent to participate in the study before taking part in any study-related activities/ procedures. Each participant will have a study number (ID) that will be used as the identifier. Only anonymised data will be collected on the questionnaires. All the information will be kept confidential. The identifiable data will only be accessed by the research team. Data protection and sharing will adhere to the Data Protection and Privacy Act 2019 of the Republic of Uganda.

An independent trial steering committee (TSC) will be set up to oversee all study activities including ensuring that the study is conducted according to the study protocol and that the participants’ safety is considered. The principal investigator will write a progress report to the TSC every 6 months, and this will be discussed during the biannual meetings and recommendations made.

The study results will be shared with participants, policymakers, other stakeholders and published in peer-reviewed journals. The results will also be communicated to the public via newsfeeds on the Makerere University Lung Institute website, and through news media outlets.

### Strengths and potential limitations

A key strength of this study is the use of cluster-randomised design that allows for objective assessment of the effectiveness of the interventions. The study will be conducted in primary care health facilities where most children and adolescents in Uganda first seek care. We will generate the much-needed information on how to improve the identification of children with asthma in low-resourced settings. The study participants will be obtained from primary care public health facilities only. The secondary and tertiary care facilities, private health facilities and other medical care points like drug shops and pharmacies will not be involved, and this will limit the generalisability of the study findings. The diagnosis of asthma will be made based on clinical features alone. LFTs such as spirometry or peak expiratory flow rate will not be done, and this may lead to underdiagnosis or overdiagnosis of asthma. Currently, the diagnosis of asthma in most health facilities in low-resource settings is made clinically due to lack of LFT equipment, supplies and technicians.^13 22 23^ Despite this limitation, we argue that the information from this study may help improve the diagnosis of asthma in settings without capacity to perform LFTs. We plan to conduct future studies in this area so that asthma care challenges can be addressed through the different types and levels of healthcare system.

## Data Availability

No data are available.
